# Emerging Approaches for the Discovery of Lipid-Based RNA Delivery Systems

**DOI:** 10.3390/pharmaceutics17091231

**Published:** 2025-09-22

**Authors:** Paul Meers

**Affiliations:** School of Environmental and Biological Sciences, 272 Foran Hall, Rutgers, The State University of New Jersey, 59 Dudley Rd., New Brunswick, NJ 08901-8250, USA; paul.meers@rutgers.edu

**Keywords:** lipid, nanoparticle, design, formulation, delivery

## Abstract

This brief review is a non-comprehensive look at some of the important aspects of lipidic nucleic acid delivery systems with a focus on RNA. In the context of this review on lipid-based formulation, nucleic acids are one of the key cargoes. Here, a brief historical background is given, highlighting a few of the key newly developing approaches to aid formulation design. These new techniques are discussed within a framework of “bottom-up” (rational) versus “top-down” (combinatorial) design. Evolving areas of interest that are discussed include multiplexed formulation and efficacy testing, new principles established in the role of the protein corona, details of the biophysical mechanism of delivery and machine learning approaches to design.

## 1. Introduction

Nucleic acid delivery is one of the most important applications of lipid-based therapeutic formulation. The field of lipid-based nucleic acid formulations, particularly lipid nanoparticles has been well documented and discussed in many excellent review articles, such as the recent extensive reviews by Wei et al. [1] and Hou et al. [2] or the historical perspective of Cullis and Felgner [3], as well as others [4,5,6,7,8,9]. The goal in this report is to provide a brief non-comprehensive overview that is partly historical, but also points out a few examples of the important new technologies that are allowing for better formulation discovery by investigating a wider scope of formulation parameters and developing a better understanding of the principles that govern delivery. The focus here is primarily on overarching principles of lipid nanoparticle formulation for RNA therapeutics, especially mRNA.

## 2. Historical Evolution of Lipid-Based Nucleic Acid Formulations

The main historical challenge for formulations of nucleic acid therapeutics has always been meeting the requirement for protection and stability of the cargo versus providing the mechanism for a pathway into the interior of the target cell where the active therapeutic must act. A delivery particle that is relatively inert and stable between the point of administration and its target must then become dynamic enough to breach the cellular barriers, such as the cytoplasmic or endosomal membrane. It is clear that this challenge requires delivery vehicles composed of multiple components and with specific adaptable physical characteristics.

**Figure 1:**
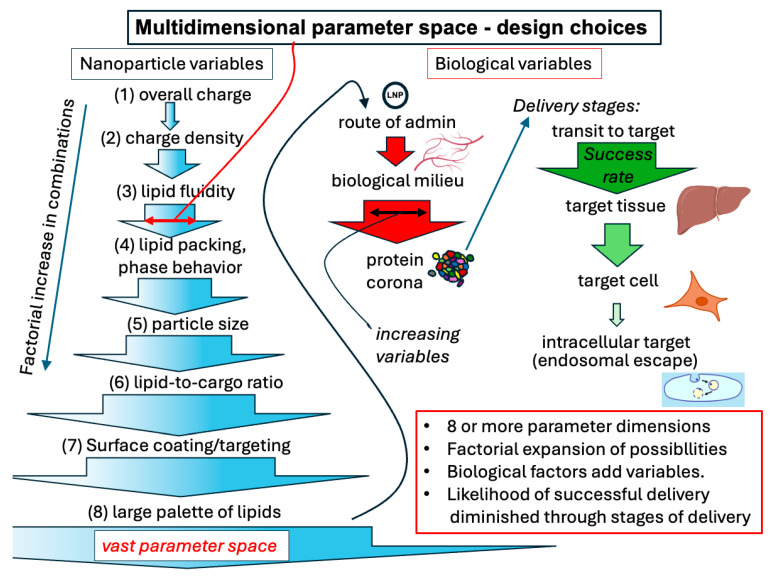
Representation of lipid nanoparticle design parameter space and evaluation. Nanoparticle variables generate a factorially increasing set of combinations of possible particles because of multiple dimensions (shown as each of 8 steps), represented on the left. Each type of particles’ delivery characteristics will depend on the route of administration and hence the protein coating obtained by the particle, represented by red arrows in the middle. The particle must then reach the target tissue, the particular target cell type, and the interior of the cell for delivery of the nucleic acid with decreasing success rates at each step, represented by green arrows on the right. As discussed in the text, endosomal escape following uptake is a particularly challenging step in this process. Nanoparticle figures adapted from and target tissue and cell icons directly from BioRender (https://biorender.com), licensed under CC BY 4.0, Mac OS version, 17 May 2025, accessed date 1 September 2025.

Two categories of approaches to find optimal formulations are to try to design these based on a relatively complete predictive understanding of the interaction of delivery vehicles with their biological milieu and targets (bottom–up approach) and/or to develop methods to rapidly and completely search and test the vast parameter space available for the design (top–down approach), recognizing that combinations of these approaches can be used (see graphical abstract). Among the many parameters that can be varied in formulation design are particle size, overall charge and charge density, lipid fluidity and packing characteristics, expected lipid phase behavior, lipid-to-cargo ratio, and the use of any combination of thousands of natural lipids or an increasing palette of synthetic lipids. There is also the possibility of accessory non-lipidic surface components to protect or target the particle. Taken together, the formulation parameter space is enormous (Figure 1). Assuming at least 1000 different lipid combination choices, which is conservative, and sampling only 10 conditions within each parameter, such as particle size, gives 10^9^–10^10^ combinations for just these eight parameters. Coupling this complexity with the complexity of various biological targets and the unknown ways in which the designed particles will interact with them dictates that better methods must be developed to explore these numerous parameters. Over the decades of lipid-based formulation development, progress has been made gradually but steadily.

**Figure 2:**
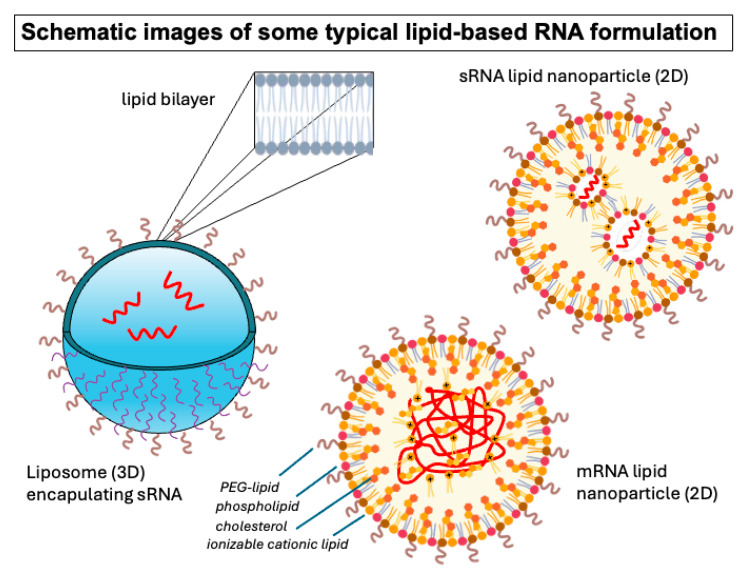
Representation of some of the lipid-based formulations used for nucleic acid delivery. Graphic representations do not capture all aspects of particle structure but offer a summary. A liposome is represented on the left in 3 dimensions. Short nucleic acids are depicted. Encapsulation occurs within the aqueous interior of the liposome without specific interaction or complexation with components of the liposome. Small RNA and messenger RNA lipid nanoparticles are represented on the right in 2 dimensions, showing the key feature of lipid–nucleic acid interactions and reduced aqueous space. Nanoparticle figures adapted from BioRender (https://biorender.com), licensed under CC BY 4.0, Mac OS version, 17 May 2025, accessed date 1 September 2025.

Nucleic acids, particularly RNA, are susceptible to degradation or immune surveillance when administered to humans [10,11]. Therefore, one of the pillars of lipid-based nucleic acid formulation design has always been protection of the nucleic acid. Historically there were many avenues available in terms of the type of particles that could be used to complex or encapsulate nucleic acids. Several types of lipid organization have been available for formulation design. Many of the earlier approaches envisioned utilization of the encapsulating liposomal technologies that were initiated long ago by Bangham and coworkers [12,13]. This contrasted with the possibility of using complexes of positively charged lipids with nucleic acids, an approach discovered by the Felgner laboratory at Stanford in the 1980s [14,15] (Figure 2).

The biological rationale for the delivery of nucleic acids encapsulated in the aqueous space of a unilamellar liposome was the expectation that a membrane fusion event could deliver an uncomplexed nucleic acid into the cytoplasm where it would be available to reach its target. However, the design of appropriately fusogenic liposomal systems that also maintained stability and protection of the cargo before reaching the intended target cell proved to be especially challenging. Moreover, loading large nucleic acids efficiently within the aqueous interior of liposomal structures without the benefit of mechanisms of locally concentrating the nucleic acids to associate with the nascent liposome made this approach even more challenging. Therefore, methods in which nucleic acids spontaneously associate with the lipids, forming a self-assembling delivery system, ultimately became the dominating paradigm, culminating in today’s lipid nanoparticles (LNPs).

LNP have the following formulation advantages:

- High (nearly 100%) complexation efficiency (w/cationic or ionizable cationic lipids);
- Rapid formulation methods (including microfluidics);
- Combinatorial synthesis of alternative (less toxic) lipids;
- Better DNA/RNA synthesis methods for stabilization;
- Easier multiplexing of formulation and testing.

Cationic lipids, such as the prototypical 1,2-Dioleoyloxy-3-trimethylammonium propane (DOTAP) or the earlier 1,2-Dioleyloxy-3-trimethylammonium propane (DOTMA), were shown to form lipid particles when complexed with DNA [14,15]. Early obstacles that had to be overcome included the fact that some complexes comprised substantial stretches of uncomplexed nucleic acid [16,17]. Optimization of charge-to-lipid ratios, the use of helper lipids and other improved formulation parameters helped to better ensure that the nucleic acid was thoroughly covered and inaccessible to nucleases or immune surveillance [17]. The development of the use of ionizable cationic lipids based on the pioneering work of the Cullis laboratory [18,19] allowed for tunable particle assembly. Furthermore, the use of polymer–lipid conjugates such as PEG–phospholipids was important to stabilize particle formation and to make both liposomal and LNP formulations more stable in biological environments [20,21,22,23,24].

A second concern for cationic lipid–nucleic acid complexes was whether there would be a mechanism of dissociation from the complex and whether it would occur in the right place and time, i.e., when the complex has reached its target. Only in recent years has an understanding of the mechanism of de-complexation within cells begun to clarify the situation. Initial studies indicated that uptake of lipid nanoparticles, followed by interaction with the endosomal membrane, appears to provide a sufficient mechanism for release of nucleic acids and transfer into the cytosol if the particle is formulated appropriately [25]. Standard lipid nanoparticles that are in use now appear to contain small aqueous spaces with individual or few nucleic acid molecules lined by lipids that are primarily cationic. When these particles interact with the endosomal membranes, PEG-lipids on the surfaces appear to disorganize and possibly transfer into the endosomal membranes, allowing for an even more avid interaction of the interior ionizable cationic lipids with anionic endosomal membrane lipids. In other words, lipid nanoparticles gradually reorganize while interacting with the endosomal membranes [25]. During this process, there apparently can be fusion-like events that allow nucleic acids to access the aqueous space of the cytosol. But the details of this process are still unclear. Recent work discussed and cited below has provided increasingly detailed and relevant mechanistic information which can form the basis for further formulation improvement.

Lastly, because cationic lipids are not generally found in nature, there had been concern about toxicity [26]. In fact, many of the early cationic lipids and their formulations did show strong toxicity to cells with which they interacted and thus could not be used in animal models. However, newer, less toxic versions were devised [27,28], and the development of multiplexed methods of lipid syntheses, i.e., combinatorial synthesis, has allowed scientists to search libraries of lipids to find less toxic cationic lipids. The development of the lipidoids by Love and coworkers [29] was an early example of this approach, which has now become common. Decades of research with the resulting less toxic ionizable cationic lipids ultimately led to a functional standard nucleic acid formulation of four lipid components in which the ionizable cationic lipids remain primarily complexed and sequestered in the interior of the particle until uptake, helping to reduce toxicity. This standard LNP has most notably led to a successful formulation for the Cominraty vaccine for COVID-19 [30].

The eventual success of this largely bottom–up approach for LNP design provided a starting point for further formulation exploration. While much has been learned about the requirements for successful nucleic acid formulations in this process, it is very likely that other parts of the formulation parameter space will need to be addressed for other future potential therapeutic targets. Faster and more comprehensive ways of searching for optimal formulations are needed, as well as a better knowledge of first principles that govern delivery in any particular biological context. In the following discussion, a few selected areas of emerging technology are addressed as some of the more important ones for advancing formulation discovery.

## 3. Emerging Technologies for Formulation Design

Several emerging technologies appear to now be aiding the search for appropriate nucleic acid formulations and deserve highlighting. These new approaches are allowing for progress from the top down using improved formulation screening methods, as well as a better understanding from the bottom up of the biological parameters that can be used to predict successful formulation design (see graphical abstract). Many of these general principles apply to small and large RNAs alike but details will vary.

The key to better screening for the best nucleic acid formulations (and other lipidic formulations as well) has essentially come down to addressing three major bottlenecks. One has been the rapid, reproducible production of well-characterized small-scale particle formulations for testing, i.e., ideally the creation of delivery particle libraries. The second issue has been the assessment of activity in relevant in vivo systems for such libraries. Lastly, an understanding of the biology of delivery, including what molecules adsorb to the surface of delivery particles and how delivery of nucleic acids into cells actually occurs, is needed to feed back into formulation design.

### 3.1. Top-Down Approach—Screening Formulations

Ideally formulation and testing technologies would allow for the creation of diverse particle libraries accompanied by methods to test these libraries in vivo. Some recent technological advances that are allowing for greater exploration of the parameter space around lipidic nucleic acid formulations include combinatorial particle formulation, e.g., [31], barcoding for analysis of the performance of multiple formulations in vivo, e.g., [32].

#### 3.1.1. Multiplexed Formulation

Inherent in the ability to discover optimized lipid-based particles has been the development of methods to rapidly and reproducibly assemble particles. The invention of mixing methods that incorporate appropriate turbulent flow with high enough Reynolds number to achieve complete mixing before initiation of particle assembly has been crucial to the advancement of the field. An early developer of this kind of technology was the Prudhomme laboratory with the invention of the flash nanoprecipitation method [33]. Mixing chambers in which impinging jets of solvent and anti-solvent containing the aqueous-soluble component or lipids, respectively, were designed for a high-Reynolds-number turbulent flow, and it was demonstrated that small, generally sub-100 nm, polymer-stabilized particles could be produced consistently when a high enough mixing rate was generated. This technology became the basis for further advances in LNP formulation, including aqueous-soluble biological polymers such as nucleic acids [34,35,36,37,38].

Extending from this pioneering work, methods of creating multiple formulations simultaneously or in a short time have been assisted by microfluidic technology. Formulation discovery requires the ability to create hundreds to thousands of combinations of components that can sufficiently cover the vast parameter space engendered in complex multicomponent systems (Figure 1). Microfluidic technology allows scientists to begin this search. Although a number of successes were reported in multiplexed formulation using early microfluidic device designs [39], most suffered from a low Reynolds number and hence slow mixing rate compared to the rate of particle formation. This led to lower uniformity and larger particle sizes [40]. Adaptations of microfluidic mixing, such as adding ridges and other internal flow obstacles for chaotic advection or using turbulent coaxial flow, led to much more rapid effective mixing rates more suitable for reproducible particle formation [41]. One widely used example of this approach is the staggered herringbone mixer [31,42]. A very detailed comparison of this configuration with a turbulent axial flow illuminates the advantages and disadvantages of each [41]. Other approaches such as acoustic micromixers can also be used to achieve more rapid mixing in microfluidic devices [43]. An excellent review by Ahl of mixing technologies for mRNA lipid nanoparticles was also published as part of this Special Issue [44]. Direct comparisons of microfluidic mixing devices are difficult because of the many formulation parameters affecting performance, but a list of informative publications is given in Table 1. It will be important to continue to develop improved mixing methods to prepare reproducible, well-characterized libraries of LNPs.

Approaches are now being designed with appropriate microfluidic devices [62,63] or other high-throughput mixing methods [63] to search the chemical/physical parameter space of LNPs by creating such libraries of particles. For instance, an approach that used a cluster-based workflow and microfluidic methods to produce particles representing appropriate regions of the parameter space followed by in vivo performance analysis led to identification of new candidate mRNA formulations for lung delivery [64].

#### 3.1.2. Multiplexed In Vivo Testing

**Figure 3:**
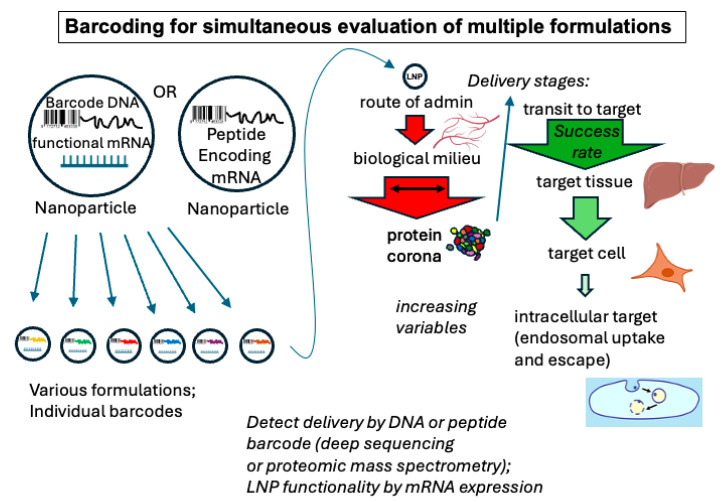
Barcoding for simultaneous evaluation/screening of multiple LNP formulations. As represented by the graphic on the left, each formulation type is associated with a specific DNA or peptide encoding mRNA barcode for identification after bioassay. Functionality of the nanoparticle formulation is assessed by expression of mRNA for a peptide or to generate a marker protein, such as the gree fluorescent protein. A group of different nanoparticles can be assessed together in vivo, comparing the performance that assessed the entire delivery process, as represented on the right. Tissue and cell icons from BioRender (https://biorender.com), licensed under CC BY 4.0, Mac OS version, 17 May 2025, accessed date 1 September 2025.

The ability to produce libraries of particles necessitates the ability to test the performance of multiple formulations in systems as close as possible to the intended clinical situation. Traditionally, in vitro testing has been a standard first step in formulation selection. Though this approach has some value, it really does not appropriately test the actual properties needed for successful therapeutic delivery. Only in vivo delivery or a validated substitute can be expected to predict efficacy. Therefore, methods to rapidly test multiple formulations in vivo are being developed. Most promising among those are the newly developed barcoding methods. In these methods, the various nucleic acid lipid nanoparticle formulations contain a barcode identifier in the form of a DNA or RNA barcode or an mRNA that codes for an identifier protein or peptide (Figure 3). In one embodiment of this principle, the Dahlman lab used mRNA for Cre recombinase as a functional expression readout via the insertion of the gene for a fluorescent protein reporter between LoxP sites in an engineered mouse strain. The fluorescent readout from this inserted gene, along with single-cell sequencing to detect specific DNA barcodes associated with specific nanoparticle formulations, allowed for the identification and assessment of specific formulations that delivered to cells to actively express mRNA-encoded fluorescent markers [65,66,67,68].

To avoid spurious barcode readouts from nanoparticles that had only adsorbed to the surface of cells, another approach has been to formulate mRNA to produce barcode peptides for particle identification. These could then be directly detected by liquid chromatography/mass spectrometry to both identify and establish functional delivery in the same event [69,70,71]. Further advances in multiplexed monitoring of the efficacy of multiple formulations can be expected to substantially advance formulation discovery.

### 3.2. Bottom-Up: Understanding Biologically Dictated Design Principles

Rational design approaches to LNPs have been undeniably successful but in an incremental way. Understanding the basic physical, chemical and biological principles that govern the efficacy of delivery can allow for an approach that builds on this knowledge. In particular, a basis of knowledge of how cationic/ionizable lipids organize into particulate structures has fueled this method of design [72,73]. But beyond the physical and structural characteristics of individual lipidic components of LNPs, a number of other parameters are now being addressed to add to design principles. As mentioned, the details of these principles will depend on the type of nucleic acid cargo.

#### 3.2.1. The Importance of the Protein Corona

**Figure 4:**
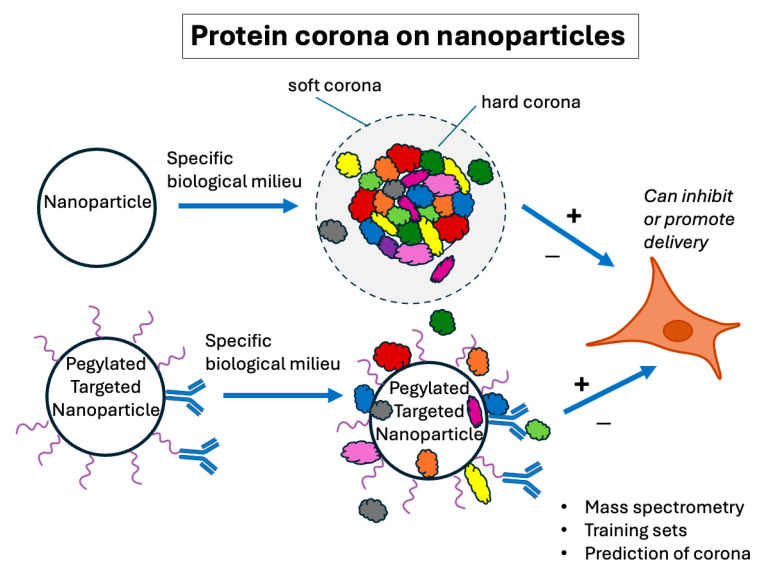
Protein coronas on lipid nanoparticles. A generic nanoparticle is represented. Multiple proteins (depicted as multiple colors) adsorb directly and relatively tightly to the surface of the nanoparticles forming the hard corona. Other less avidly bound proteins form the soft corona. Nanoparticle surface coatings, such as PEG (pegylated), depicted in the lower scheme, tend to lessen but not eliminate the adsorbed proteins. Targeting moieties, such as ligands or antibodies, can help target the nanoparticle but are sometimes inactivated by the protein corona. Cell icon from BioRender (https://biorender.com), licensed under CC BY 4.0, Mac OS version, 17 May 2025, accessed date 1 September 2025.

It has been known for many years that liposomes and lipid nanoparticles (and, in fact, all delivery particles) invariably collect some sampling of proteins from their physiological environment before they reach their intended therapeutic target. Opsonization of liposomes was described as early as the 1980s [74,75]. Only recently has it become possible to rapidly and efficiently monitor the composition of this protein coating, now referred to as the protein corona (Figure 4). As the corona represents the exposed surface of the particle, it can dictate the particle’s in vivo behavior. It is now known that identifiable “hard” and “soft” coronas can exist [76,77], where the former refers to the proteins directly and tightly bound to the particles and the latter refers to less tightly bound outer layers of protein that are often washed off of particles recovered from incubations in biological milieu (Figure 4). These proteins and the native or denatured conformations they adopt on nanoparticle surfaces can dictate the targeting and delivery characteristics of nanoparticles. It has become clear that an operational understanding of the inevitably associated corona is crucial.

Advances in technology to simultaneously identify large cohorts of polypeptides have allowed scientists to begin to better understand the corona and how it affects delivery. In particular, proteomic liquid chromatography/mass spectrometry (LCMS) is an advance that has allowed for the identification of large groups of proteins based on a comparison of the mass-to-charge ratio of thousands of tryptic peptides to the predicted molecular weights from genomic sequences. As discussed in excellent reviews by Mahmoudi et al. [78] and Bashiri et al. [79], LCMS has been used to analyze the population of protein molecules that have become associated with various nanoparticles, including liposomes or LNPs after contact with specific biological milieu [78,80]. In some cases, nanoparticles have even been used as tools to sample and identify the blood plasma proteome [81,82]. Bound proteins can influence LNP performance positively or negatively even when they are formulated with surface polymers designed to minimize protein adherence, such as PEG [76,83], and when targeting moieties, such as antibodies or ligands (which will not be discussed here), are attached to the particle surface [84,85]. Although surface PEG inhibits the total amount of protein adsorption in general, there is still a distinct adsorbed proteome that influences LNP efficacy [86]. A further complication is the fact that coronas are biofluid-specific [80] and those obtained from in vitro incubation are significantly different from those in vivo [87].

Discoveries such as these have led to the prospect of using the corona proteome to actually promote delivery. Manipulating the protein corona, such as through the elimination of opsonins, can dramatically change the behavior of nanoparticles [88]. In fact, specifically monitoring and manipulating the protein corona on nanoparticles can potentially be used as a method of targeting delivery [85,86,89,90,91,92]. For instance, Chen et al. [91] found that LNP compositions could be manipulated to specifically modulate the adherence of apolipoproteins and vitronectin, resulting in an enhancement in delivery. Another example of this effect was presented by Miao et al. [93]. Though ApoE is known to be a major adsorbed serum component targeting nanoparticles to the liver, proteomic analysis of the nanoparticle hard corona showed that the combination of ApoE and serum albumin is more important in determining the targeting of the nanoparticle. Accumulated data on the role of the corona proteome in delivery can eventually be used to generate training sets for predictive analysis through machine learning (see below).

#### 3.2.2. Imaging, Spectroscopic and Computational Methods to Understand LNP/Liposome Interactions with Cells

One other key area of technological development that has allowed for a better understanding of the principles that govern delivery has been the recent ability to observe the disposition of individual nanoscale particles in biological samples. Although there have long existed methods of monitoring lipid-based formulations using radioactive or fluorescent labeling, it has only recently become possible to go below the Abbe diffraction limit to observe the mechanisms by which lipid-based particles interact with their target (or non-target) cells in non-fixed tissue. Liposomes and lipid nanoparticles are typically smaller than 200 nm and can be as small as tens of nanometers. When observed with visible light, the diffraction limit typically does not allow particles smaller than hundreds of nanometers to be separately distinguished [94]. This means that all optical methods of observation of lipid nanoparticles, including advanced methods such as confocal laser scanning microscopy, have a limit of resolution larger than the particles themselves.

Transmission electron microscopy has been used to reach subnanometer resolution but generally requires harsh sample preparation conditions in which lipid particles do not survive well or that distort the relevant images. The advent and development of cryo-electron microscopy has allowed scientists to obtain high-resolution images of flash-frozen samples with minimal use of harsh contrast agents. An example of the importance of cryo-electron microscopy comes from the imaging of LNP formulations as isolated particles. In particular, there is substantial evidence from cryo-EM and molecular dynamics simulations that siRNA and oligonucleotide formulations often have an internal structure comprising RNA-containing compartments with similarity to inverted HII-phase lipid organization [95,96,97] (Figure 3). Small-angle X-ray and cryo EM methods have been used to establish a structure–activity relationship for LNP-formulated antisense oligonucleotides [98], where such structural features are also observed. Yap et al. [99] recently showed a correlation between complex LNP internal structures such as cubosomes and delivery efficiency in CHO cells.

It is not clear whether the much longer strands of protein-coding mRNA would actually show such an organization in the LNPs. In fact, the properties and delivery mechanisms of mRNA LNP could be considerably different from formulations with short polynucleotides with the possibility of heterogeneity in the lipid distribution in the interior of the mRNA-bearing particles [100,101,102]. Additionally, siRNA duplexes, for example, would lack the complicating factor of possible secondary structure formation that would exist for some mRNAs during formulation. Nonetheless, both siRNA and mRNA (1922 bases) have been reported to form internal cubic phase structures within certain LNPs with enhanced in vitro cellular delivery compared to LNPs with lamellar internal structures [103]. Faceted external bilayer structures in phytosterol-containing LNPs have also been correlated with efficiency of delivery [104]. Involvement of non-bilayer structures within the LNP in the ultimate delivery mechanism of some LNPs would not be surprising considering that particular lipids or conditions that lead to inverted hexagonal phases have long been known to be associated with fusogenicity [105,106]. Other LNP structural correlates have also been investigated, such as the apparent association between mRNA-rich “bleb” structures and LNP delivery activity [107]. An excellent overall review of LNP structure has recently been published [45].

Additionally, computational methods for coarse-grained simulated structure and pKa prediction have been developed for lipid nanoparticles [108,109]. These studies appear to give some degree of consensus to the correlation of non-bilayer phases with LNP delivery efficiency. Simulations with short mRNAs also show a pH-dependent transition to similar structures [110], but do not address most much longer protein-coding mRNA formulations. Aside from structure alone, Parot et al. [111] compiled orthogonal analyses of a number of nanoparticle physical characteristics to correlate with in vitro delivery efficiency. This analysis primarily addressed how to validate data for comparative analysis. Although the delivery outcomes assessed were in vitro, the framework for comparative analysis of quality LNP parameters can be important for establishing datasets that can be useful for machine learning approaches to LNP design, which are discussed below.

**Figure 5:**
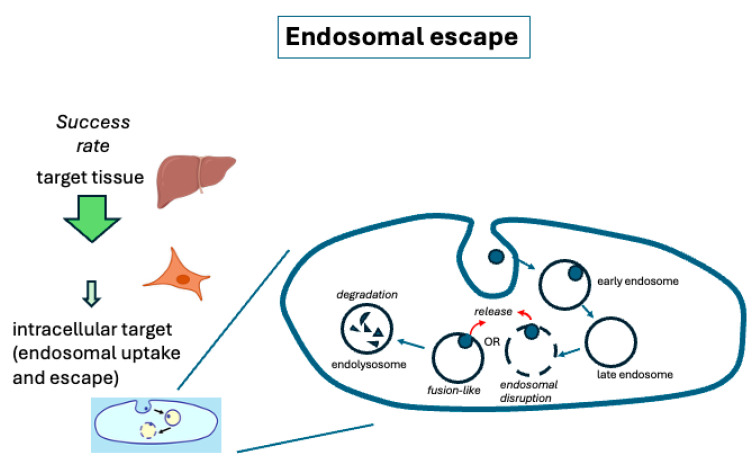
Generalized cellular uptake pathway for lipid nanoparticles. The cell uptake graphic on the right illustrates possible fates of a nanoparticle taken up by cells. Release or escape must occur before the particle is degraded in a later endolysosomal compartment. Cell and tissue icons from BioRender (https://biorender.com), licensed under CC BY 4.0, Mac OS version, 17 May 2025, accessed date 1 September 2025.

Although isolated LNP particle parameters can be predictive, even more useful principles may emerge from observing or modeling the actual interactions between LNPs and the target membrane. LNPs typically undergo a process of internalization into target cells by one of several possible pathways, most commonly leading to several different compartments, including one called the endosome. The particle must escape the endosome to bring cargo into the cytosol of the cell before it is delivered to later compartments, such as the endolysosome, where degradation occurs (Figure 5). Endosomal compartments are known to be a barrier to delivery of nucleic acids as many observations of accumulation of lipid nanoparticles in the endosomal pathway have been reported, while the efficiency of endosomal escape has been estimated to be approximately 1–2% in most cases [112,113,114,115]. But the details of the direct interaction of LNPs with endosomal membranes has been difficult to study. A more complete understanding of the mechanistic details of nucleic acid delivery across the endosomal membrane is still needed. However, recent new methods are beginning to provide more important information, as in the examples presented below.

Super-resolution fluorescence microscopy, which allows for observation of non-frozen samples to resolutions down to approximately 20 nm, has been applied to observe the behavior of individual mRNA LNP in their transit through the endosomal uptake pathway [116]. This study revealed nucleic acid separation from LNP and entry into the cytosol at the stage of early endosomes. Recent studies of mRNA LNP interactions with supported bilayers representing a very simplified endosomal membrane mimic have revealed possible aspects of the nucleic acid delivery across the endosomal membrane [117]. One notable result was the release of much of the mRNA into what would be the endosomal lumen, followed by rebinding to the surface of the model endosomal membrane where disruption could subsequently allow for movement into the model cytosolic compartment. Such a mechanism could be consistent with the inefficiency of delivery. Real-time SAXS analysis has been used to follow nanoparticles containing cubosome or hexosome structures as they interact with vascular endothelial cells [118]. These studies revealed a structural transition within the particles driven by the interaction with the endothelial cells. Computational molecular dynamics simulations have also shown some possible mechanisms of endosomal escape [119]. Coarse-grained molecular dynamics of a model short double-stranded DNA LNP predicted distinct modes of interaction depending on the orientation of the DNA helix with respect to the modeled endosomal membrane. This simulation also predicted facile release of the DNA after a slower initiation of transfection. A recent molecular dynamics study provided further insight on RNA-LNP, elucidating aspects such as transient and dynamic internal LNP structures and lipid migration under low-pH conditions with correlation with observed delivery activity [120]. Better information on the details of this mechanism of delivery will be crucial to help guide the design of lipid nanoparticles.

#### 3.2.3. Machine-Learning-Based LNP Design

Machine learning design of small-molecule drugs and novel proteins has seen an explosion of activity. Applications such as protein folding have succeeded with robust and well-defined datasets, i.e., the use of protein structures from X-ray, NMR and EM studies to correlate to sequences where there is little ambiguity about comparisons of data. Large language model approaches to lipid nanoparticle design are now also being developed, as reviewed by Dorsey et al. [121] and others [122], but are limited by the kinds of training datasets available. Additionally, the complexity of the multiple hierarchical parameters in even the simplest lipid nanoparticle and the large number of possible ways in which to search the multiparameter space that governs their activity means that only small parts of that space are being explored at this point. A further constraint is that many of the available training datasets measure transfection efficiency in terms of in vitro systems, potentially lacking predictive power for in vivo delivery. The consistency of the physicochemical properties of the assessed formulations can also be a concern. For instance, particles of exactly the same composition can vary in efficiency depending on other formulation parameters (such as mixing rates) that might not have been controlled.

Nonetheless, within the framework of the now relatively standard four-component lipid nanoparticles, it has been possible to adapt machine learning algorithms to assess the applicability of structural variants of individual lipid components, particularly the ionizable lipid [123,124,125,126,127,128,129,130]. The power of the approach is illustrated by two of the top-performing new lipids that were predicted using lipid optimization via neural networks (LiON), a deep learning method [126]. FO-32 and FO-35 had unexpected unique chemical structures representing a chemical space not previously explored. In some cases, such as in the work of Cheng et al. [131], parameters beyond individual lipids were assessed. These included compositional ratios and general lipophilicity of the helper lipid, albeit using in vitro data. Bae et al. [127] modeled 7 general compositional parameters, along with 307 individual lipid substructure fingerprints, using data from 213 different mRNA LNPs evaluated by intradermal injection. From this training data, important structural components of ionizable lipids were predicted by this model in terms of transfection efficiency, immune response and formulation characteristics. Most recently, Kumar et al. presented machine learning methods based on 6454 different LNP formulations and importantly included analyses to try to bridge the gap between in vitro and in vivo results [132].

Target cell phenotypic features have also been exploited as training sets to understand and identify relevant targets for LNP delivery optimization. Cell biological features were used by Hunter et al. [133] as datasets to predict the most appropriate cellular uptake targets for successful delivery. A dataset of varying efficiencies for delivery of mRNA-coded mCherry by a standard LNP formulation was created by treating a model cell line with a large number of siRNA and small-molecule inhibitors that were expected to affect delivery. Rather than correlating with the expected targets of the inhibitors, the authors created a training dataset for Random Forest and Boosted Tree analysis from advanced image analysis of a number of different characterized markers for uptake, such as fluorescent 70 kDa dextran. Machine learning analysis of the cell images and corresponding LNP-mCherry mRNA expression efficiency led to insights about optimal cellular uptake targets, with the macropinocytosis pathway revealed as a top candidate.

Particle structure is another developing area in which datasets can be used to train computational algorithms to predict delivery efficacy. As outlined above, a number of studies have already explored these correlations. In fact, commercial cryo-EM image analysis products are now being offered to assess LNP properties for quality control and to predict efficacy. These approaches may allow high-throughput analysis of LNP formulation attributes to establish predictive structural features. Ultimately, delivery particle libraries to generate large training datasets along with large language model machine learning approaches may be the most productive path to predicting and exploring the unknown in lipidic particle design. As more data is accumulated and standards are established, it may be possible to extend into unexplored formulation types.

Another area of application for predictive algorithms can be based on the role of the protein corona. There has now been enough proteomic data compiled on corona proteins to begin to develop training sets to try to predict corona compositions using large language models [134,135]. However, at this point, the studies have been largely performed for other types of delivery systems such as metal nanoparticles [136] or DNA nanostructures [79]. As pointed out [78], the training data comes mainly from the protein characteristics rather than the particle characteristics, demonstrating the ongoing need for suitable training datasets based on nanoparticle formulation characteristics. Ideally, in the future, a corona composition could be predicted from formulation parameters with delivery characteristics then based on the expected LNP-associated proteome.

## 4. Conclusions

The discovery of lipid particle formulations for nucleic acids has now gained a number of useful new tools that can potentially aid design. Exploring more of the design parameter space and a better understanding of the basic principles underlying the delivery mechanism will be the needed drivers of better performance. The ability to produce large libraries of nanoparticles and barcoding methods to evaluate them, especially in vivo, will allow for exploration of a substantially greater range of potential formulations. New information on the mechanisms of nanoparticle delivery at the molecular level, including super-resolution microscopy and methods like SAXS, may allow the basic governing principles to be better understood, while defining and understanding the effects of the array of proteins which adhere to the particles will help to inform directions in design. It will be interesting to see in the coming years how far beyond the currently standardized LNP formulations these new methods will lead us. With the extremely complex biological attributes of various potential therapeutic targets, it is likely that the new territory explored by these evolving methods will lead to newly accessible therapeutic targets.

## Figures and Tables

**Table 1:** Information for comparison of microfluidic devices for LNP preparation.

Device	Source of Relevant Information	General Comment
T-junction/Y-junction	[44,45,46,47,48,49,50,51]	The type of mixer that has a low Re, long mixing time, higher polydispersity
Hydrodynamic flow focusing (HFF/3-inlet flow focusing)	[41,44,46,48,49,50,51,52,53,54]	faster mixing, can dilute samples
Staggered herringbone mixer (SHM)/ chaotic micromixers	[38,39,40,41,42,44,46,47,48,49,50,51,53,54,55,56,57]	Faster mixing, can clog
Ring/toroidal micromixer	[44,51,53,58,59]	Faster mixing, can clog
3D/multi-layer microfluidic focusing (vertical focusing VFF, multi-inlet), vortex	[46,50,51,60,61]	Faster mixing
Droplet (emulsion) microfluidics → solvent removal	[50,51]	
Acoustic/electrokinetic micro-mixers (on-chip active mixing)	[43,46,50,54]	Faster mixing

## Abbreviations

The following abbreviations are used in this manuscript:

LNP	Lipid nanoparticle
LCMS	Liquid chromatography/mass spectrometry
EM	Electron microscopy
